# Oxidation Mechanism of Al-Sn Bearing Alloys

**DOI:** 10.3390/ma14174845

**Published:** 2021-08-26

**Authors:** Qiaoqin Guo, Jihui Chen, Jianping Li, Yongchun Guo, Zhong Yang, Wei Yang, Dapeng Xu, Bo Yang

**Affiliations:** 1 School of Material Science and Engineering, Xi’an Technological University, Xi’an 710032, China; chenjihui1108@sina.com (J.C.); yc-guo@126.com (Y.G.); yz123456@126.com (Z.Y.); weiy1106@126.com (W.Y.); pengd1003@126.com (D.X.); 2 Jiangyin Micro-Arc Metal Technology Co., Ltd., Jiangyin 214431, China; li_ht@126.com

**Keywords:** Al-Sn bearing alloy, oxidation mechanism, oxidation kinetics, precipitation

## Abstract

Oxidation of Al-Sn bearing alloy occurs during production, processing and use, which reduces both alloy performance and performance of coatings applied to the alloy surface. Therefore, the oxidation mechanism of Al-Sn bearing alloy is studied at 25, 180, 300, and 500 °C. The oxidation morphologies of the alloy were observed by scanning electron microscopy (SEM), and the oxidation products were determined by X-ray diffraction (XRD). The oxidation weight gain curves were obtained by thermogravimetric analysis. The experimental results show that: Al-Sn bearing alloy is oxidized quickly to form Al_2_O_3_. As the oxidation temperature increases, Sn phase start to precipitate along the grain boundary and form networked spheroids of Sn on the alloy surface. The amount of precipitation increases with further increase of the oxidation temperature. Cracks and holes are left in the alloy. The oxide layer is mainly composed of Sn, SnO_2_, and Al_2_O_3_. At 25 °C, oxidation rate of Al-Sn alloy approach zero. At 180, 300, and 500 °C, the oxidation rate increases quickly conforming to a power function, and eventually remains stable at about 3 × 10^−6^ mg·mm^−2^·s^−1^.

## 1. Introduction

Engine is the key component of automobiles, tanks, armored vehicles [1], ship, and other equipment, in which the bearing bush is a very important part in the engine to protect the spindle and connecting rod [2,3,4]. Al-Sn bearing alloys have good anti-seizure property and fatigue strength [5,6,7,8]. In order to obtain Al-Sn bearing alloy with better performance, Cruz et al. [9], studied the effect of Al-Sn bearing alloy casting microstructure on the final mechanical properties and wear resistance. They concluded that increasing the primary dendrite arm spacing improved wear resistance. Wang et al. [10], studied the effect of ultrasonic wave on solidification of Al-Sn-Si alloy, and reported that ultrasonic wave promoted formation of uniform microstructure and control of the grain size. Lu et al. [11], studied the wear behavior of Al-12 wt % Sn alloy filled with nano-Si/Sn composite, and found that this composite had excellent tribological properties. However, with the continuous improvement of the speed and load of the modern engine, the adverse effects of wear under working conditions are being increasingly recognized as a potential technological limiting factor. The alternating stress increases greatly, especially when the engine starts or accelerates instantaneously. Under such conditions the lubricating oil film cannot be formed in time, and the crankshaft and bearing bush are in dry grinding state, which can lead to the easy damage of the bearing bush and crankshaft, resulting in engine scrap [12,13]. Therefore, the bearing bush must additionally have excellent anti-friction performance [14]. The traditional plating of double-layer and three-layer bearing bush cannot meet the requirements of its use. Therefore, using surface treatment technology to prepare antifriction coating on the surface of bearing alloy is the focus of current research on bearing alloy materials [15,16]. Non-equilibrium magnetron sputtering ion plating technology has been widely used to prepare coatings with fine grain size, uniform structure, and good friction resistance [17,18]. However, the magnetron sputtering process is complex. Before preparing the coating, the workpiece needs to be pretreated by removing surface impurities and oxide layer. Moreover, the coating is very sensitive to surface treatment and fails easily when the treatment is improper. It is well known that Al surface is easily oxidized; so does the Al-Sn bearing alloy. Its oxidation increases with increase in oxidation time. The oxidation film constitutes a barrier between the anti-friction film and the Al-Sn alloy, leading to poor adhesion, coating failure, and subsequently, failure of the bearing shell [19,20].

Therefore, before depositing anti-friction coating, the oxide film must be removed from the surface of Al-Sn bearing alloy, so as to obtain the coating with good comprehensive performance and improve the service life of the bearing shell.

Peng et al. [21] studied the constant temperature oxidation kinetics of 4004 Al alloy surface, and that reducing the heating temperature during hot rolling and controlling the thickness of the oxide layer on the alloy surface could improve the success rate of hot rolling composite of three-layer aluminum alloy (4004/3003/4004). Yuan et al. [22] studied the high-temperature oxidation behavior of rare-earth Al alloys and found that the rare-earth-rich phase precipitate along the grain boundary of the low-rare-earth alloy. Due to its preferential oxidation, the diffusion channel of the grain boundary is blocked and the oxide grain size changes. Eventually, leading to a reduction in the oxidation rate of Al. Yu et al. [23] studied the regular transformation of Al oxide during the oxidation of Al at high temperature, and found that due to the presence of molten Al in the core of the oxidized Al powder particles, the presence of molten Al destroyed the core-shell structure and also promoted the shrinkage of the shell. This causes liquid Al to diffuse into the shell, and promotes oxygen to diffuse into the shell.

However, there is no systematic study on the oxidation kinetics of Al-Sn bearing alloy on the surface of bearing bush. Therefore, the oxidation kinetics of Al-Sn bearing alloy was systematically studied, which lays a theoretical and empirical foundation for the processing and forming of bearing alloy, the preparation of high-performance coating by magnetron sputtering and the use and protection of bearing alloy.

## 2. Experimental Methods

In this study, an Al-Sn bearing alloy with the size of 10 × 10 × 2 mm, composed of 10# cast steel with an Al-Sn alloy on its surface, 10# steel is a kind of steel material with good plasticity and toughness, and is easy to cold and hot forming. Its implementation standard is GB/T699-1999. Two alloys were combined to form a double alloy layer (Al-Sn bearing alloy) by stamping, and then annealed to eliminate residual stress and homogenization of microstructure. The thicknesses of the steel and Al-Sn alloy were 10 mm and 350 μm, respectively. The composition of the Al-Sn alloy was shown in Table 1 and the chemical composition of 10# steel was shown in Table 2.

Oxidation furnace was used for the oxidation experiment of Al-Sn alloy. The Thermo Gravimetric Analyzer (HTG-DZ-1, Beijing Henjiu Experimental Equipment Co. Ltd., Beijing, China) was used to research the oxidation kinetics. Since the Al-Sn alloy may be placed at 25 °C for a long time before processing and its working temperature was 180 °C, the oxidation temperatures were set to 25 °C and 180 °C. The oxidation temperatures were also set 300 °C and 500 °C to study the oxidation rules of the Al-Sn alloy at higher temperature. The oxidation time was 12 h, and the Thermo Gravimetric Analyzer was used to record the weight every 0.2 s automatically.

Using the Formulas (1) and (2) to calculate the oxidation rate of the Al-Sn alloy

(1) $$G=\frac{{\Delta g-(S}_{1}\text{×}v\text{×}t)}{S_{2}}$$

(2) $$V=\frac{G}{t}$$

where △g is the weight gain of the steel/Al-Sn alloy, g; G is the weight gain per unit area of alloy, g·mm^−2^; v is the oxidation rate of the 10^#^steel, g·mm^−2^·s^−1^, to determine the oxidation rate of steel, it is necessary to conduct a separate oxidation experiment on the steel at the same temperature, and then use Formula (2) to calculate v; S_1_ is the surface area of steel in the steel/Al-Sn alloy, mm^2^; and S_2_ is the surface area of the Al-Sn alloy in the steel/Al-Sn alloy. V stands for the oxidation rate of Al-Sn alloy.

The surface and cross-sectional morphologies of the oxidized Al-Sn alloys were observed via scanning electron microscopy (VEGA·II XMU, TESCAN, Brno, Czech Republic) at 35 kV, and the phase compositions of the samples were analyzed by X-ray diffraction (XRD-6000, Shimadzu, Tokyo, Japan), scan range was between 20–90° and scanning speed was 2°/min at a scan step of 0.020°. In order to study the effect of oxidation on the matrix, SEM was used to observe the surface morphology after removing the oxide layer.

## 3. Results and Discussions

### 3.1. Microstructures of Al-Sn Alloy

**Figure 1:**
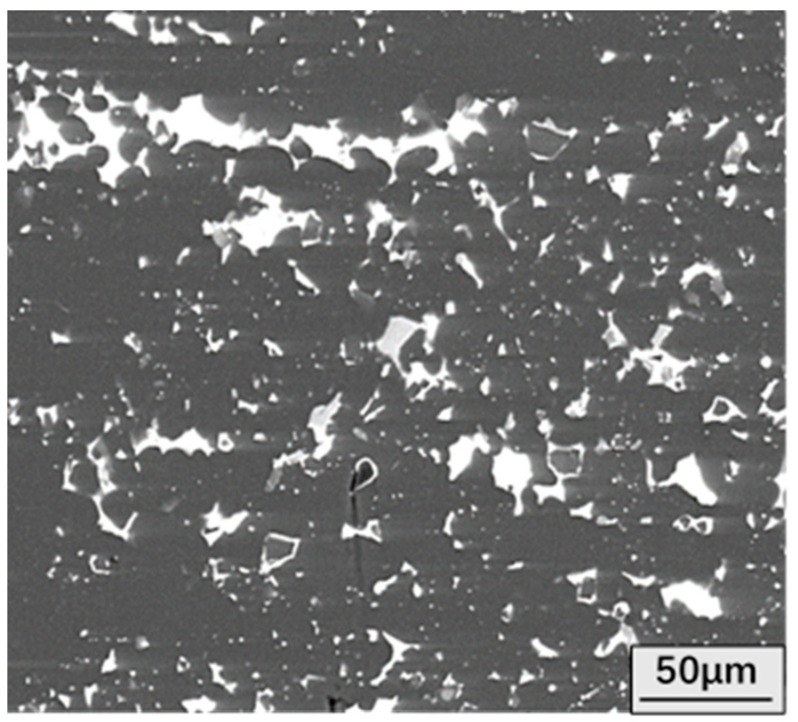
Microstructures of the Al-Sn alloy.

**Figure 2:**
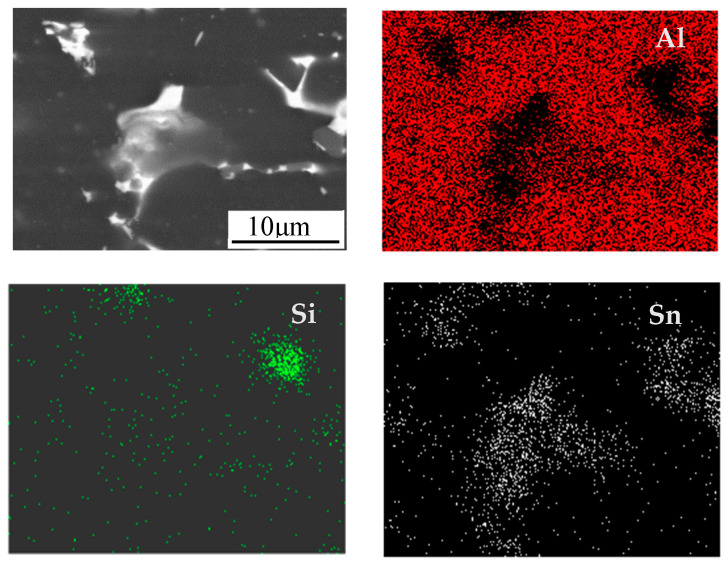
Surface SEM images and EDS map of Al-Sn alloy oxidized for 12 h at 25 °C.

In order to compare with the surface morphology of the oxidized alloy, the surface morphology of the unoxidized alloy is first studied. Prior to surface characterization, the sample is ground and polished, and then SEM imaging and EDS (Energy dispersive scanning technique) elemental detection and analysis are performed. The unoxidized microstructure of Al-Sn alloy is shown in Figure 1. Al-Sn alloy is a monotectic alloy [24]. The atomic radius of Al is 143 pm, and the atomic radius of Sn is 158 pm. According to Hume–Rothery rules, ΔR = (R_Sn_ − R_Al_)/R_Al_ = 10.5%, close to 15%, the atomic radii of Al and Sn is significantly different, and it is difficult for Sn to dissolve in Al. Al-Sn alloys are sometimes called mutual insoluble alloys. The white granular Sn phase is continuously and uniformly distributed in the dark gray Al matrix. Figure 2 shows surface SEM images and EDS map of Al-Sn alloy oxidized for 12 h at 25 °C. Sn and Si are uniformly distributed on the whole, but there is a local aggregation in the gully formed by Al matrix. Zhang et al. [24] found that Sn is distributed on the grain boundary of Al in granular form. However, in the alloy ingot just produced, Sn is distributed in the grain boundary in a network structure, and the degree of uniformity is low. After heat treatment, it reaches a relatively uniform level, but it leaves some voids generated during heat treatment [25]. The addition of Sn can improve the tribological properties [26], but also reduce the hardness of aluminum alloy. Si is added to improve the hardness of Al-Sn bearing alloy, which leads to improvements in the tribological properties [27].

**Figure 3:**
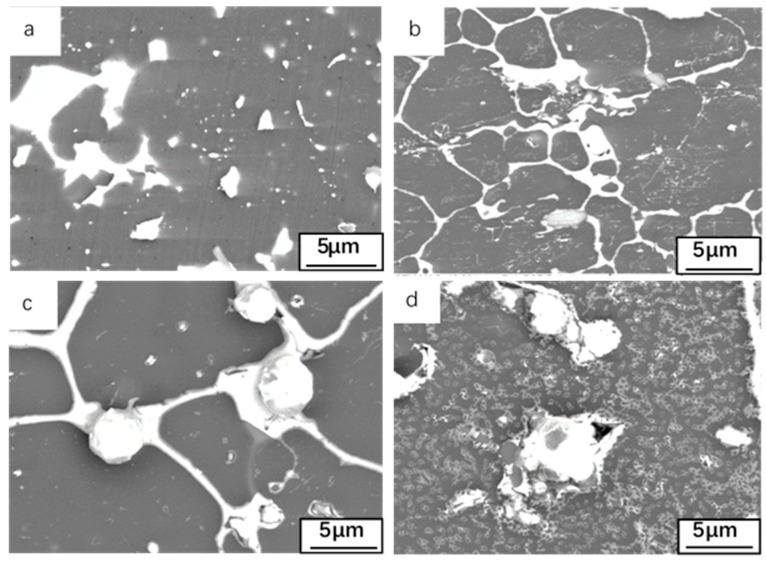
Surface morphologies of oxide films formed after 12 h at different temperatures (**a**) 25 °C, (**b**) 180 °C, (**c**) 300 °C, (**d**) 500 °C.

Surface morphologies of oxide films formed after 12 h at different temperatures as shown in Figure 3. In the formation process of Al-Sn bearing alloy, in order to make Sn dispersed in the alloy, it is often necessary to undergo heat treatment, but this will make part of the vacancies generated in the heat treatment process be retained, resulting in the vacancy in the Al-Sn bearing alloy in the supersaturated state. The vacancy binding energy of Sn is large, so Sn atom is easy to combine with the free state vacancy [28], while the free state vacancy significantly affects the low-temperature diffusion rate of solute atoms, so the diffusion rate of Sn in matrix Al is fast [29]. With the help of vacancy migration, Sn also slowly precipitates from the matrix in the form of elemental at 25 °C. As the temperature gradually increases, Sn phases in the form of spheres and networked spheres form on the surface of the Al-Sn alloy. With the temperature being raised to 180 °C, some gases in the matrix gather at the α-Al triangular grain boundaries. There are channels between the α-Al grains [30]. When the temperature is higher than the melting point of Sn, 232 °C, Sn in liquid state will flow into the channels. The volume of Sn after melting increases (the density decreases), making the liquid Sn flow directly from the matrix. In addition, when the gas precipitates, Sn phase will be excluded. When the channel mouth is located on the surface of the alloy, it also promotes Sn to form spherical Sn phase [31]. Formation of spherical Sn phase is favored because it can reduce the interfacial energy per unit volume in the nucleation process, and the nucleation is easier. The higher the temperature, the greater precipitation. With the oxidation temperature increasing, the degree of surface oxidation is intensified, and the oxide film Al_2_O_3_ formed on the surface will become less adherent. In addition, the surface stress of the oxide film is also redistributed [32], and the cracks generated on the surface are more conducive to the precipitation of Sn. There are some holes after Sn distributed on the surface is precipitated as shown in Figure 3d. The distribution of Sn with network structure only occurs at 180 °C and 300 °C, and agglomeration occurs at 500 °C.

**Figure 4:**
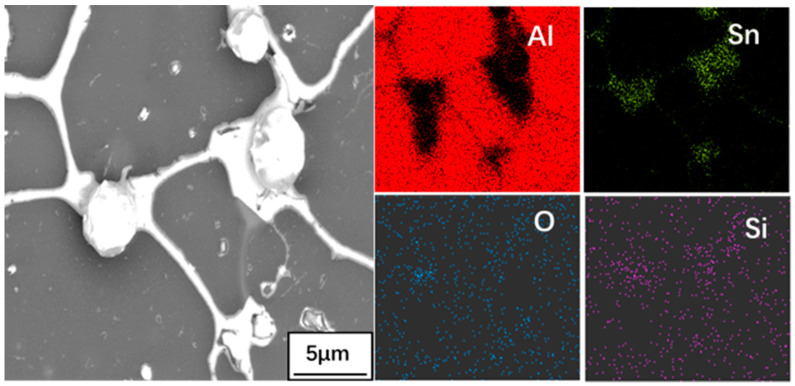
Surface SEM images and EDS map of Al-Sn alloy oxidized for 12 h at 300 °C.

Surface SEM images and EDS map of Al-Sn alloy oxidized for 12 h at 300 °C is shown in Figure 4. Sn precipitates along the grain boundary and channels between α-Al grains. Then, Sn will form network and spherical Sn phases respectively. It can be seen from Figure 4 that the O element is mainly distributed in the area where the network Sn phase forms on the alloy surface. Outside the network Sn phase region, O element distributes uniformly. It indicated that part of Sn element is oxidized to SnO_2_. Al is oxidized in a very short period of time, so an oxide layer Al_2_O_3_ that protects the bearing alloy layer is formed on the surface of Al-Sn bearing alloy. This layer effectively impedes oxidation of the substrate. The main reaction equation is: Sn + O_2_ = SnO_2_, 2Al + 3O_2_ = 2Al_2_O_3_.

**Figure 5:**
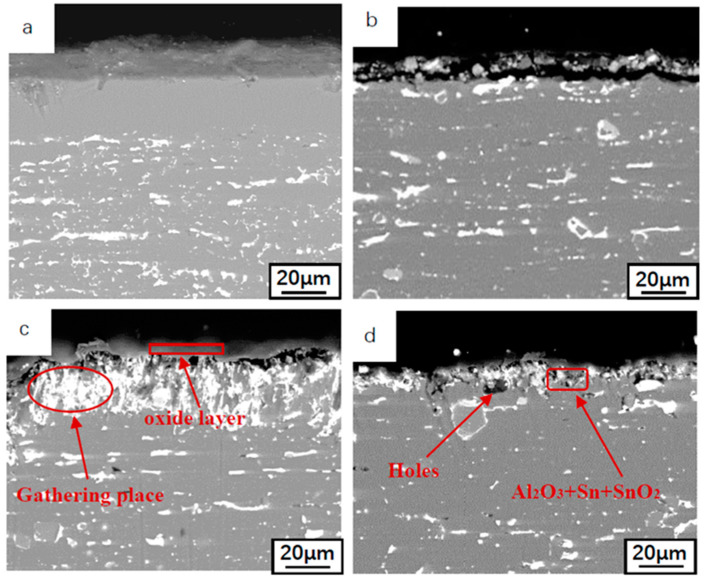
Cross-sectional morphologies of the oxide films at different temperatures (oxidized for 12 h) (**a**) 25 °C, (**b**) 180 °C, (**c**) 300 °C, (**d**) 500 °C.

Figure 5 shows the cross-sectional morphology of Al-Sn alloy oxidized at different temperatures for 12 h. The distribution of Sn in the matrix changes little when the alloy is oxidized at 25 °C. Under these conditions, Sn can only diffuse to the surface in the form of atoms. With the increase of temperature, a large amount of Sn precipitates from the Al-Sn bearing alloy layer towards its outer surface. When temperature exceeds the melting point of Sn, Sn flows directly from the diffusion channel to the surface in liquid form. The higher the oxidation temperature, the less residual Sn in the matrix. After oxidation at 500 °C for 12 h, most of Sn precipitated in the matrix. The higher the oxidation temperature, the thicker the oxide layer formed on the surface. In addition, the Sn phase accumulated under the layer will gradually precipitate with the increase of the oxidation temperature and leave some voids in the original aggregation.

**Figure 6:**
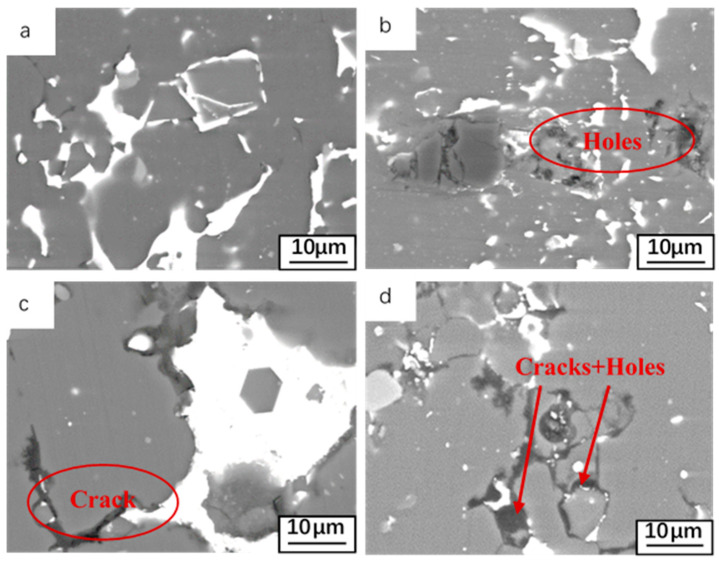
Microstructures after removing oxide film at different temperatures: (**a**) 25 °C; (**b**) 180 °C; (**c**) 300 °C; (**d**) 500 °C.

In order to study the effect of oxidation on the alloy matrix, it was necessary to perform some pre-treatment before SEM imaging. This was accomplished by first removing the oxide layer on the oxidized alloy surface with sandpaper, and then polishing the oxidized alloy surface before SEM imaging. Figure 6 shows the surface morphology of the oxide layer that has been oxidized at 25, 180, 300, and 500 °C for 12 h. For sample oxidized at 25 °C (Figure 6a) no changes were observed in the distribution of Sn on the surface. When the oxidation temperature is 180 °C, except for the oxide layer on the surface of Al-Sn alloy, Sn aggregation can be seen on the surface of the alloy. In addition, because a small amount of Sn has been precipitated, some cracks and holes will remain in the lower seam. When the oxidation temperature is 300 °C, the aggregation of Sn is more obvious on the surface of the alloy removed the oxide layer, but the distribution of Sn on the surface of the alloy is extremely uneven. The increase of temperature increases the diffusion driving force of Sn. At 300 °C or even higher than the melting point of Sn, Sn becomes liquid will also provide volume-increasing tension, so Sn is easier to precipitate. When the temperature is increased to 500 °C, almost all the aggregated massive Sn phases are precipitated from the surface of Al-Sn alloy, so a large number of cracks and holes are left on the surface of the alloy.

### 3.2. Phase Composition Analysis

**Figure 7:**
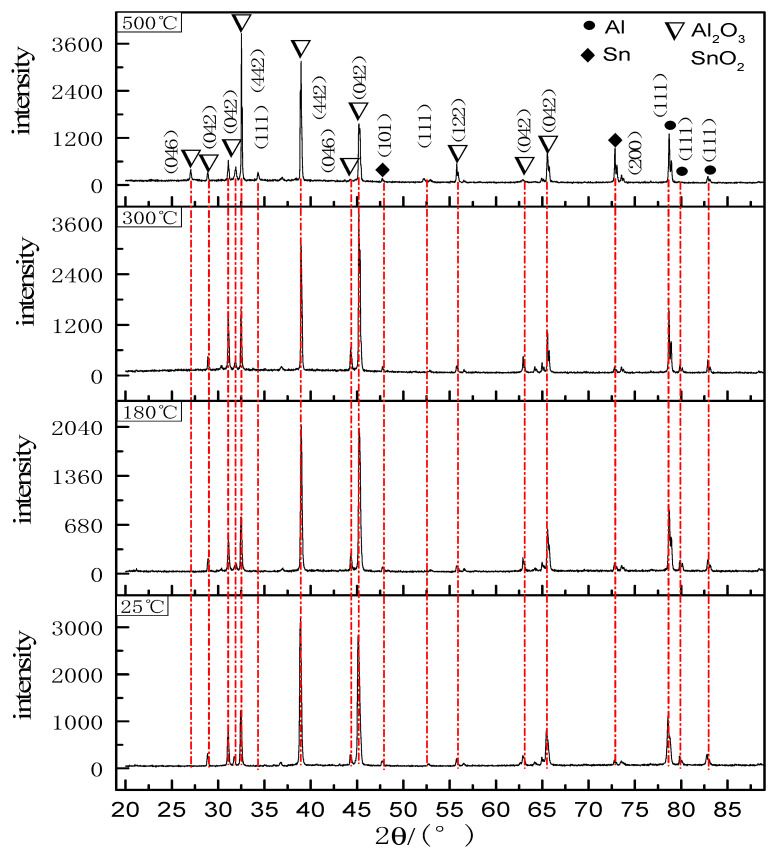
XRD spectra of oxide film at different temperatures (t = 12 h).

XRD data was acquired from the sample that has been subjected to oxidation experiment in the oxidation furnace. Figure 7 shows the XRD pattern of Al-Sn bearing alloy after oxidation at 25, 180, 300, and 500 °C for 12 h. From Figure 7, it can be observed that after 12 h of oxidation at different temperatures, the phases present remain unchanged, namely Sn, SnO_2_, Al, and Al_2_O_3_. Sn mainly grows preferentially along the (101) and (200) planes. The preferred growth planes of Al_2_O_3_ are (046), (042), (422). The preferred growth surface of SnO_2_ is (111). As the temperature increases, the diffraction peak intensity of Al is almost unchanged. The reason is that the oxide layer is relatively thin and X-rays hit the transition layer of Al. The diffraction peaks of Al_2_O_3_ increase with the increase of temperature. In addition, the increase of temperature promotes the growth of Al_2_O_3_ along the new preferred surface. The intensity of the diffraction peak of Sn also increases with the increase of temperature, which is mainly due to the large amount of Sn precipitation caused by the increase of temperature. The intensity of the diffraction peak of SnO_2_ is very low when the temperature is below 500 °C, which indicates that Sn in the matrix mainly exists in the form of elemental tin, and it will be oxidized to SnO_2_ after precipitation. When the temperature reaches 500 °C, a small amount of Sn will be oxidized to SnO_2_. Both SnO_2_ and Al_2_O_3_ formed by oxidation have a protective effect, which can effectively prevent continuous oxidation. Therefore, it can be concluded that the oxide layer is mainly composed of Sn, SnO_2_, and Al_2_O_3_, and the related reaction equation is: Sn + O_2_ = SnO_2_, 4Al + 3O_2_ = 2Al_2_O_3_.

### 3.3. Oxidation Kinetics

**Figure 8:**
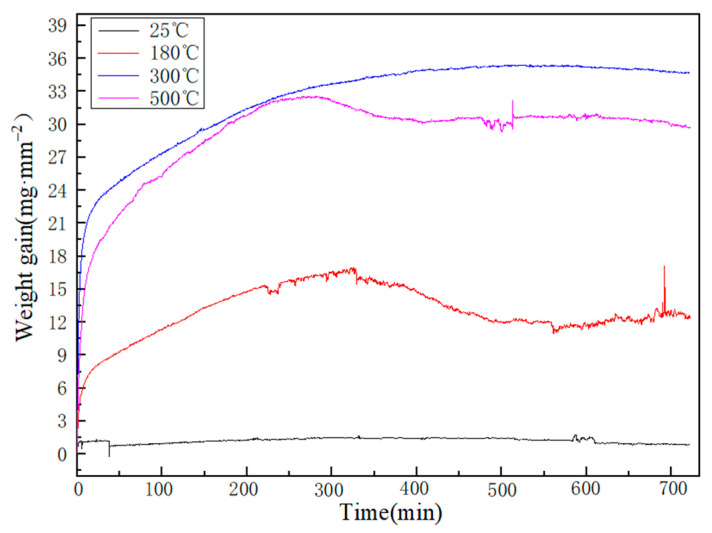
Oxidation kinetics curves of Al-Sn alloys at different temperatures.

**Figure 9:**
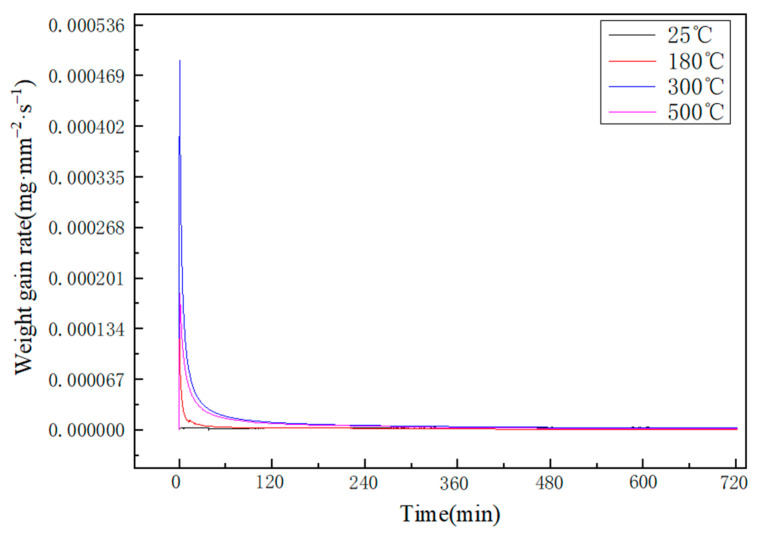
Oxidation rates of Al-Sn alloys at different temperatures.

The weight gain curves of Al-Sn alloy oxidized at different temperatures for 12 h are shown in Figure 8. The oxidation weight gain curve of 25 °C is almost a horizontal straight line. At this temperature, a very thin oxide layer of Al_2_O_3_ will be formed on the surface. Due to the low temperature, although the oxidation time increases, the oxidation does not increase. Comparing the oxidation weight gain curves at 180, 300, and 500 °C, it can be found that the weight increase of Al-Sn bearing alloy is relatively large when it enters the oxidation stage. However, over time, oxidation weight gain began to become small and eventually stabilized. Since the passivation layers with different thicknesses formed on the alloy surface at different temperatures can effectively prevent oxygen from entering, it is difficult for the oxidation to continue. The weight gain curve in the figure shows a decline, because the precipitates of the Sn phase are detached from the surface. At 500 °C, the Sn phase is easily melted and flows out of the diffusion channel, and is also the easiest to detach from the surface, resulting in a reduction in the oxidized weight gain. Therefore, the overall weight gain curve will be lower than the oxidation weight gain curve at 300 °C. Figure 9 shows the oxidation rate curve of Al-Sn alloy oxidized at 25, 180, 300, and 500 °C for 12 h. The oxidation weight gain curve of Al-Sn alloy at 25 °C is almost a horizontal straight line, so the oxidation rate is almost 0. It is oxidized at the other three temperatures. When Al-Sn alloy first enters the oxidation stage, the oxidation rate is very high, but it decreases steeply in a short time. The oxidation temperature is high, therefore, an oxidation film of Al_2_O_3_ is formed in a short time, which prevents the alloy from continuing to oxide. The final oxidation rate tends to be about 3 × 10^−6^ mg·mm^−2^·s^−^^1^.

The fitting equation for the oxidation kinetics curve of the Al-Sn alloys is shown in Table 3. The fit of the fitting equations at different temperatures is different. The oxidation rate is the largest at the beginning of oxidation. Then, the oxidation rate tends to stabilize with the oxidation time increasing and it shows that the equation has a limit value. The fit of 300 °C is the highest, because the difference between the increased weight and the reduced weight caused by the shedding of the skin is the smallest at this temperature. The fitted curve conforms to the power function curve law of oxidation kinetics.

**Figure 10:**
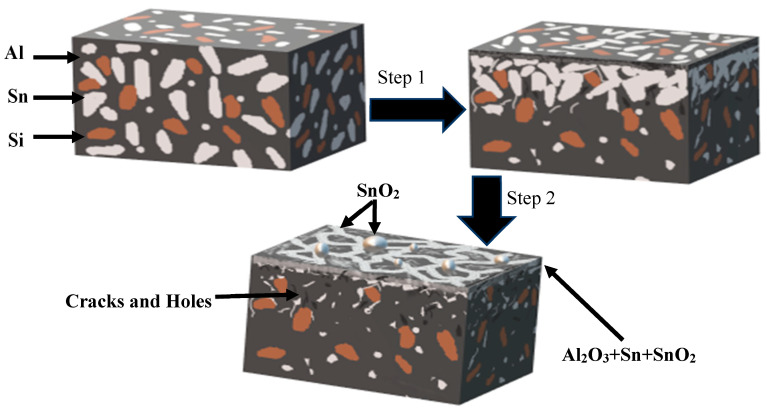
Oxidation mechanism picture of the Al-Sn bearing alloy.

In order to analyze and summarize the changes in the oxidation process of Al-Sn alloy, we draw the oxidation mechanism diagram of Al-Sn bearing alloy as shown in Figure 10. At different temperatures, the changes of Al-Sn bearing alloy during oxidation are also different, but they are accompanied by the precipitation of Sn. The precipitation of Sn is roughly divided into two stages, the first stage is the aggregation of Sn, and the second stage is the precipitation of Sn. When the temperature is 180 °C, the precipitation of Sn is in the first stage. Sn mainly occurs on the surface of Al-Sn alloy, but a small amount of Sn precipitates to form spherical and network Sn phases. When the temperature reaches 300 °C and 500 °C, the precipitation of Sn is mainly in the second stage, but the precipitation of Sn at 500 °C is more complete than that at 300 °C, and the residual Sn content in the alloy is less. When the oxidation temperature reaches 300 °C and 500 °C, the temperature has exceeded the melting point of Sn. At this point, Sn will exist in the channel of the substrate in the form of liquid. When the outlet of the channel is located on the surface of the oxide layer, the tension and gas occupation effect formed by the volume increase of Sn phase transformed into liquid promote the direct outflow of liquid Sn and the formation of spherical Sn phase on the surface. In addition, Sn will also precipitate along the grain boundary by using the vacancy diffusion mechanism to form a network Sn phases on the alloy surface. The network-like and spherical Sn formed on the surface of Al-Sn bearing alloy at 300 °C are more than those at 180 °C. When the temperature increases to 500 °C, the network-like Sn phase will disappear, but the number of spherical Sn phase is more and the volume is larger. In addition, a small amount of SnO_2_ will be formed at 500 °C. The higher the temperature, the greater the Sn precipitates, so more pits and cavities will be left in the original place of Sn after oxidation. The oxide layer has some cracks on the surface due to the redistribution of surface stress after the oxidation.

## 4. Conclusions

Al_2_O_3_ is generated on the surface of Al-Sn bearing alloy at 25 °C. When the oxidation temperature increases, the alloy is further oxidized and the thickness of the oxidized layer increases. At 180 °C and 300 °C, Sn precipitates along the grain boundary through the diffusion channel, and forms spherical and reticular Sn phases on the alloy surface. A small amount of Sn will be oxidized to SnO_2_ at 500 °C. SnO_2_ and Al_2_O_3_ both can effectively prevent continuous oxidation. The amount of precipitation increases with the increase of oxidation temperature. After Sn phase precipitation, there appear cracks and holes in the alloy. The oxide layer is mainly composed of Sn, SnO_2_, and Al_2_O_3_. At 25 °C, the oxidation rate of the Al-Sn alloy is a line approaching zero, indicating that the oxidation speed is very slight; with the increase of oxidation temperatures, the oxidation rates increase quickly conformed to power functions and eventually remain stable at about 3 × 10^−6^ mg·mm^−2^·s^−1^.

## Figures and Tables

**Table 1:** Chemical composition of Al-Sn bearing alloy.

Elements	Sn	Si	Al
Content (wt %)	5.9	4.0	balance

**Table 2:** Chemical composition of 10# steel.

Elements	C	Si	Mn	Ni	Cu	Fe
Content (wt %)	0.12	0.37	0.65	0.25	0.20	balance

**Table 3:** Fitting equations of the oxidation rate curves of the Al-Sn alloy.

Temperature/°C	Fitting Equation	Fit/R^2^
25	y = 0.0000002	0.9931
180	y = 0.0001X^−0.769^	0.9243
300	y = 0.0006X^−0.839^	0.9961
500	y = 0.0006X^−0.917^	0.9753

## Data Availability

Data from this review can be made available upon request to the corresponding author after executing appropriate data sharing agreement. The corresponding websites are listed in the manuscript.
